# 16S rRNA Amplicon Sequencing of Urban Prokaryotic Communities in the South Bronx River Estuary

**DOI:** 10.1128/MRA.00182-20

**Published:** 2020-05-28

**Authors:** Eugenia Naro-Maciel, Melissa R. Ingala, Irena E. Werner, Allison M. Fitzgerald

**Affiliations:** a Liberal Studies, New York University, New York, New York, USA; b Sackler Institute for Comparative Genomics, American Museum of Natural History, New York, New York, USA; c Biology Department, College of Staten Island, City University of New York, Staten Island, New York, USA; d New Jersey City University, Jersey City, New Jersey, USA; Georgia Institute of Technology

## Abstract

Biodiversity monitoring is an essential component of restoration efforts. We sequenced 16S rRNA gene amplicons from sediments and waters of Hunts Point Riverside Park and Soundview Park, located in a historically degraded but recovering urban estuary in New York. In total, 16,165 unique amplicon sequence variants were recovered, and *Proteobacteria* was the dominant phylum.

## ANNOUNCEMENT

With increased urbanization comes the recommendation to monitor disrupted ecological communities and maintain sustainable environments. Much of the biodiversity in New York City, New York, remains to be described, including cryptic prokaryotic communities in the South Bronx River Estuary. Here, 16S rRNA gene amplicons from 33 samples collected at Hunts Point Riverside Park (*n*_sediment_, 9; *n*_water_, 8) and Soundview Park (*n*_sediment_, 8; *n*_water_, 8) are presented. Soundview (40.81N, 73.87W) is among the successful regional oyster restoration sites (1), and Hunts Point (40.82N, 73.88W) is a former illegal dump area being revitalized as a Bronx River Greenway component.

Sampling occurred between August 2015 and September 2016, monthly from May to October during low tide. Soundview samples were collected along the restored oyster reef, an area containing live Crassostrea virginica. Surface water samples were obtained by horizontally submerging a 1-liter autoclaved jar. Core sediment samples weighing approximately 100 g were collected from the surface sediment layer with a polyvinyl chloride pipe and a pallet shovel (2). Samples were placed in a cooler and then a laboratory refrigerator and were processed within 24 h of collection. Water samples were filtered using Whatman cellulose nitrate sterile filters, and a randomly selected 0.25-g soil subsample was used for extraction. DNA was extracted with the PowerWater and PowerSoil kits for water and soil, respectively (Qiagen, USA), and quantified with a NanoDrop 2000 spectrophotometer (Thermo Scientific, USA). Samples were stored at −20° C until transfer to MR DNA (Shallowater, TX), where the bacterial V4 hypervariable region segment was amplified with the 16S primers 515F-Y (3) and R806 (4) using the HotStarTaq Plus master mix kit (Qiagen, USA) under standard conditions and controls (5). After agarose gel checks, 3 replicates of the same DNA extract were pooled in equal proportions and purified with calibrated Ampure XP beads (Agencourt Bioscience, USA) (5). A DNA library was created following the Illumina TruSeq protocol, and 16S sequencing was performed using v. 3 chemistry (2 × 250 bp) on the Illumina MiSeq platform.

In total, 4,673,520 raw reads (Hunts Point: *n*_sediment_, 1,130,030, and *n*_water_, 1,200,424; Soundview: *n*_sediment_, 1,363,728, and *n*_water_, 979,338) were sequenced and then processed with QIIME2 using default parameters unless otherwise specified (v. 2019.10) (6). The DADA2 plugin was used to quality filter, denoise, and join paired-end reads (6, 7). Sequences were truncated at 260 bp for quality purposes and trimmed to remove primers. After DADA2 filtering, 2,585,025 (per-sample average, 78,334; standard error, 73,923) or 58% of reads were retained across all samples (Table 1). The QIIME2 naive Bayesian q2 classifier was used for taxonomic classification in comparison to the SILVA 16S rRNA database (8, 9). The amplicon sequence variant (ASV) feature table, taxonomy, and sample metadata were exported to BIOM format for analysis in R (v. 3.6.1) with default parameters (https://www.r-project.org/) using the phyloseq v. 1.29.0 (10), vegan v. 2.5-6 (https://cran.r-project.org/package=vegan), and ggplot2 v. 3.2.1 (11) packages.

**TABLE 1:** Summary of sample data

Sample^*a*^	Barcode	SRA accession no.	Recovery of reads per sample^*b*^		% recovery
			Pre-QC	Post-QC	
S.B.BRO	TCCGTGCT	SRR11096228	118,335	70,435	0.60
S.B.HP	TCCGTGCG	SRR11096233	104,747	62,346	0.60
S.C.BRO	TCCGTTTT	SRR11096225	160,377	83,918	0.52
S.C.HP	TCCGTTCC	SRR11096227	149,596	88,181	0.59
S.D.BRO	TCCTCCCT	SRR11096226	168,964	105,792	0.63
S.D.HP	TCCTAATA	SRR11096224	228,336	52,539	0.23
S.E.BRO16	TCCTCGTG	SRR11096251	184,366	94,186	0.51
S.E.HP16	TCCTCGTA	SRR11096252	113,250	65,777	0.58
S.F.BRO16	TCCTGGCA	SRR11096253	100,614	51,923	0.52
S.F.HP16	TCCTGTAC	SRR11096250	125,101	69,269	0.55
S.G.BRO16	TCCTTCCA	SRR11096254	178,795	78,983	0.44
S.G.HP16	TCCTTCTT	SRR11096249	122,171	67,039	0.55
S.H.BRO16	TCCTTGGT	SRR11096248	366,360	230,102	0.63
S.H.HP16	TCGAAAGG	SRR11096245	89,237	48,264	0.54
S.I.BRO16	TCGAACCT	SRR11096244	85,917	53,166	0.62
S.I.HP16	TCGAAGAG	SRR11096246	90,311	46,350	0.51
S.J.HP16	TCGAATCC	SRR11096247	107,281	64,108	0.60
W.B.BRO	TCCGGTAA	SRR11096255	123,727	76,269	0.62
W.B.HP	TCCGGCCG	SRR11096256	156,627	103,485	0.66
W.D.BRO	TCCGTCGG	SRR11096243	130,923	90,783	0.69
W.D.HP	TCCGTCTT	SRR11096231	354,017	74,594	0.21
W.E.BRO16	TCGACCGG	SRR11096241	89,971	58,539	0.65
W.E.HP16	TCGACAAC	SRR11096242	98,081	63,046	0.64
W.F.BRO16	TCGACGAA	SRR11096237	103,653	66,728	0.64
W.F.HP16	TCGACCTA	SRR11096238	127,273	69,419	0.55
W.G.BRO16	TCGACGAC	SRR11096239	106,508	67,959	0.64
W.G.HP16	TCGAGCCG	SRR11096236	95,728	67,378	0.70
W.H.BRO16	TCGAGCGA	SRR11096240	93,017	68,157	0.73
W.H.HP16	TCGAGCTG	SRR11096235	106,190	60,540	0.57
W.I.BRO16	TGGTAACC	SRR11096234	146,998	97,546	0.66
W.I.HP16	TGGTACAA	SRR11096230	134,451	93,281	0.69
W.J.HP16	TGGTAGAT	SRR11096232	128,057	91,577	0.72
W.J.SVP16	TGGTAGTC	SRR11096229	184,541	103,346	0.56

a Samples are labeled as per Fig. 1.

b QC, quality control.

After removal of contaminant mitochondria and chloroplast sequences, 508,352 sequences and 16,165 unique ASVs were recovered, and rarefaction analysis showed sufficient sampling depth. The mean diversity indices were higher in sediment (observed ASVs: Hunts Point, 1,283.3, and Soundview, 1,646.5; Shannon diversity: Hunts Point, 6.33, and Soundview, 6.14; Pielou’s evenness: Hunts Point, 0.876, and Soundview, 0.848) than in water samples (observed ASVs: Hunts Point, 612.6, and Soundview, 734.1; Shannon diversity: Hunts Point, 4.69, and Soundview, 5.08; Pielou’s evenness: Hunts Point, 0.732, and Soundview, 0.763). The communities were dominated mostly by the bacterial phyla *Proteobacteria*, *Epsilonbacteraeota*, *Cyanobacteria*, *Bacteroidetes*, *Actinobacteria*, and *Acidobacteria*, but other phyla were present in significant proportions (Fig. 1). These analyses provide a baseline for further characterizing and monitoring the microbial biodiversity in a complex urban estuary.

**FIG 1:**
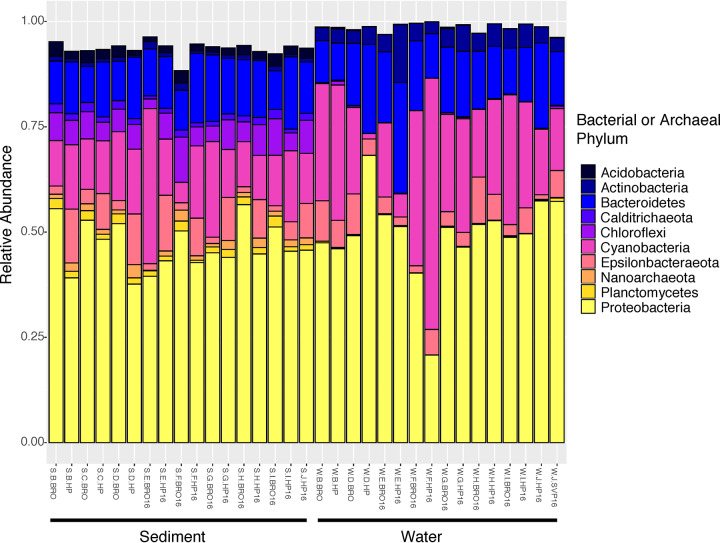
Relative abundance bar plot of the top 10 microbial phyla obtained from 16S rRNA sequencing of water (W) and sediment (S) samples at Hunts Point Riverside (HP) Park or Soundview (BRO, SVP) Park. Each color shade represents a different phylum of bacteria or archaea, and sample type is indicated by bars below the graph. Samples collected in 2016 have “16” included in the name, while all other samples were collected in 2015. All organisms belong to the domain *Bacteria*, except for the *Nanoarchaeota*, which is classified in *Archaea*.

### Data availability.

The 16S rRNA amplicon gene sequences from this study have been uploaded to the GenBank Sequence Read Archive (SRA) under the BioProject accession number PRJNA606795 (Table 1).
